# Global analysis of seasonal changes in trematode infection levels reveals weak and variable link to temperature

**DOI:** 10.1007/s00442-023-05408-8

**Published:** 2023-06-26

**Authors:** Rachel A. Paterson, Robert Poulin, Christian Selbach

**Affiliations:** 1 Norwegian Institute for Nature Research, Torgarden, PO Box 5685, 7485 Trondheim, Norway; 2 Department of Zoology, University of Otago, PO Box 56, Dunedin, 9054 New Zealand; 3 Department of Arctic and Marine Biology, UiT The Arctic University of Norway, Langnes, PO Box 6050, 9037 Tromsø, Norway

**Keywords:** Freshwater ecosystems, Global warming, Latitude, Parasitism, Phenology, Summer, Transmission

## Abstract

Seasonal changes in environmental conditions drive phenology, i.e., the annual timing of biological events ranging from the individual to the ecosystem. Phenological patterns and successional abundance cycles have been particularly well studied in temperate freshwater systems, showing strong and predictable synchrony with seasonal changes. However, seasonal successional changes in the abundance of parasites or their infection levels in aquatic hosts have not yet been shown to follow universal patterns. Here, using a compilation of several hundred estimates of spring-to-summer changes in infection by trematodes in their intermediate and definitive hosts, spanning multiple species and habitats, we test for general patterns of seasonal (temperature) driven changes in infection levels. The data include almost as many decreases in infection levels from spring to summer as there are increases, across different host types. Our results reveal that the magnitude of the spring-to-summer change in temperature had a weak positive effect on the concurrent change in prevalence of infection in first intermediate hosts, but no effect on the change in prevalence or abundance of infection in second intermediate or definitive hosts. This was true across habitat types and host taxa, indicating no universal effect of seasonal temperature increase on trematode infections. This surprising variation across systems suggests a predominance of idiosyncratic and species-specific responses in trematode infection levels, at odds with any clear phenological or successional pattern. We discuss possible reasons for the minimal and variable effect of seasonal temperature regimes, and emphasise the challenges this poses for predicting ecosystem responses to future climate change.

**Supplementary Information:**

The online version contains supplementary material available at 10.1007/s00442-023-05408-8.

## Introduction

Seasonal cycles are among the most important drivers of ecosystem dynamics on ecological time scales (Fretwell 1972). The predictable changes in temperature, daylight, and other abiotic factors that accompany seasonal shifts are the main cues of phenological responses in individuals, triggering and synchronising processes such as plant flowering or the onset of animal migrations and reproductive activities (Forrest and Miller-Rushing 2010; Varpe 2017). At higher levels, seasonal shifts also act as key regulators of population and community dynamics, and therefore, they determine temporal fluctuations in ecosystem functions and services (Tonkin et al. 2017; White and Hastings 2020).

Yet, it remains difficult to make ecosystem-wide generalisations regarding the responses of individuals or populations to seasonal transitions. One reason for this is that even among closely related taxa, idiosyncratic responses are not uncommon. For instance, aquatic insects show species-specific patterns of emergence phenology and do not all respond to temperature in the same way (Finn et al. 2022). Another reason is that research effort in this area has been uneven across higher taxa and geographical regions (Woods et al. 2022). For example, in freshwater ecology, the phenology of several taxa, such as molluscs, non-planktonic crustaceans, and non-salmonid fish, has received relatively little attention despite these taxa playing important roles in their communities and ecosystems (Woods et al. 2022). Entire functional groups are often left out of classical studies of seasonal community succession. In particular, parasites rarely feature in such studies, despite evidence showing that the dynamics of parasitic diseases are strongly influenced by seasonal transitions (Altizer et al. 2006).

Temperate freshwater ecosystems have been particularly well studied in terms of seasonal cycles, with forces ranging from physical factors to nutrient limitation now understood to shape the typical successional patterns of key players such as phyto- and zooplankton from spring through summer and into autumn (Sommer et al. 2012). Yet, seasonal successional changes in the relative or absolute abundance of different components of aquatic ecosystems are also incompletely resolved due to a lack of knowledge regarding certain key taxa. Trematode parasites are one such taxon. Not only are these endoparasitic flatworms very diverse in many lakes and rivers, but they can also account for substantial biomass and productivity that seem disproportionate to their small body sizes and cryptic nature (Preston et al. 2013; Lagrue and Poulin 2016). Both their free-swimming infective stages and parasitic stages can be important prey items for various consumers in aquatic food webs (Morley 2012; Thieltges et al. 2013). Trematodes can impact individual hosts, for example by castrating them or increasing their mortality rate (e.g., Kelly et al. 2010; Marchand et al. 2020), as well as impact population dynamics and community processes (Bernot and Lamberti 2008; Friesen et al. 2020), especially under thermal stress (Mouritsen et al. 2018). Thus, incorporating their seasonal cycles of abundance and infection levels within the broader successional framework developed for freshwater ecosystems is needed for a more complete and holistic understanding of these systems and how they will respond to future climate change.

Although there is some variation among species, the typical trematode life cycle involves three host species, two phases of direct transmission (or environmental transmission), and one of trophic transmission (Galaktionov and Dobrovolskij 2003). First, a miracidium hatched from an egg infects a mollusc (usually a gastropod) first intermediate host, in which it will undergo several rounds of asexual multiplication to produce free-swimming infective stages known as cercariae. Second, after emerging from the mollusc host, cercariae seek and penetrate their second intermediate host (an invertebrate, tadpole or fish, depending on the trematode species), in which they encyst as metacercariae. Third, the life cycle is completed when an infected second intermediate host is ingested by a suitable vertebrate definitive host, in which the parasites will develop into adult worms and produce eggs that will be released in host faeces.

Another reason why trematodes must be considered in the context of seasonal community changes in freshwater systems is their notorious sensitivity to temperature. Small changes in temperature can have profound effects on the infectivity of miracidia (Morley and Lewis 2015), the rate at which cercariae are produced asexually in the molluscan host (Poulin 2006), the survival and infectivity of cercariae to the second intermediate host (Morley 2011; Morley and Lewis 2015), as well as parasite-induced mortality of all hosts in the life cycle (Paull and Johnson 2011; Friesen et al. 2021). However, as suggested before (Marcogliese 2001; Rohr and Cohen 2020), the responses of aquatic parasites to temperature are likely to be idiosyncratic and more nuanced than indicated by laboratory studies. There is indeed evidence that universal seasonal patterns in trematode infections of freshwater hosts may be rare. For example, within-mollusc cercarial production rates and their subsequent survival after leaving the mollusc in response to changing temperatures show much interspecific variation, even among related and/or sympatric trematode species using the same mollusc host species (Selbach and Poulin 2020; Born-Torrijos et al. 2022; Taskinen et al. 2022). Similarly, although infection of fish serving as either second intermediate hosts or definitive hosts are often high during summer months, there is huge variability in seasonal patterns among species of freshwater fish or trematodes (Poulin 2020). The factors driving these species-specific responses, and what they mean for the integration of seasonal patterns of trematode infections within the broader community succession framework for freshwater systems, remain to be addressed.

The main goal of this study is to test for general seasonal patterns of infection by trematodes in temperate freshwater ecosystems, and identify the key drivers of variation in their seasonal responses. An earlier study (Poulin 2020) based strictly on fish hosts and using crude winter-versus-summer contrasts indicated that seasonal changes in trematode infections are highly variable. Here, we extend this approach to all stages of the trematode life cycle and use a more precise metric of local seasonal temperature changes to test for global patterns of seasonality in trematode infections. Specifically, we evaluate the impact of the local thermal regime, the habitat type, the host taxon, and the host’s role in the life cycle (first intermediate host, second intermediate host, or definitive host) on the magnitude of changes in trematode infections from spring to summer. To achieve this, we have assembled and analysed a large global-scale dataset derived from published field studies across multiple host taxa. Our results will allow trematode seasonality to be incorporated with known successional patterns in freshwater systems. They also add a new layer of complexity and realism to current attempts to anticipate ecosystem changes in disease risk in the face of climate change.

## Methods

### Data compilation

We compiled a dataset from published studies by searching the Web of Science in June 2022, using the following search string: (trematod* OR digenea*) AND (freshwater* OR lake* OR lentic* OR aquatic OR reservoir* OR pond* OR river OR water) AND (season* OR temporal* OR temperat* OR succession* OR summer OR winter OR spring OR autumn). The search returned 907 articles, of which 11 could not be accessed on the Internet or through any of our institutional libraries. The remaining 896 were individually examined to determine whether they met our inclusion criteria (see below). In the end, 99 articles were retained and contributed to the dataset (see full list of source articles in Supplementary Table S1).

Since our focus was on the seasonal progression of trematode infections in freshwater ecosystems from spring to summer, to be included a study had to provide data on prevalence (proportion of individual hosts infected), intensity (mean number of parasites per host, excluding uninfected ones), and/or abundance (mean number of parasites per host, including uninfected ones) of infection by one trematode taxon in one host species in one locality, over a continuous temporal cycle from spring to late summer. We included both studies that provided data on a month-by-month basis, and those that reported only data pooled by season. In the case of studies reporting data month-by-month, we excluded a few entries for which the suitable monthly data (e.g., April or May for North Hemisphere spring) were unavailable. Where data values were not directly reported in the main text or tables, values were extracted from figures using either DataThief (https://datathief.org) or ImageJ (Schneider et al. 2012). In addition, the host sample size per month or season had to be given, or at least could be estimated from total sample size. Finally, since seasons in the tropics are characterised by changes in precipitation (i.e., rainy versus dry seasons) rather than temperature, we included only studies conducted at latitudes above 25° north or south.

Each entry in our dataset consisted of a seasonal series of infection values by one trematode species in one host species in one locality and in one seasonal/annual cycle. Since some papers provided data collected on two or more parasite or host species, in more than one locality, or in more than 1 year, many papers contributed two or more entries to the dataset. For each entry, we recorded the following information: (i) trematode species; (ii) host species; (iii) host higher taxon, i.e., mollusc, crustacean, insect, leech, amphibian, or fish; (iv) whether the host served as first intermediate host, second intermediate host, or definitive host of the parasite; (v) the name of the study locality and its latitude and longitude, with the coordinates obtained from Google Maps if necessary; (vi) the habitat type, i.e., pond, river or lake (including reservoir); (vii) the year of sampling; (viii) the infection measure, i.e., prevalence, intensity or abundance; (ix) infection values for each month or season, from late winter/early spring to the following late summer/autumn; (x) the host sample size for each month or season, sometimes estimated by dividing total sample size by the number of samples; and (xi) the article in which the data were published. Because of the seasonal mismatch between the northern and southern hemispheres, data from the southern hemisphere were shifted by 6 months (i.e., southern November was matched with northern May) to achieve a seasonal match with northern localities and facilitate subsequent calculations and graphical summaries.

Additional data were obtained from other sources. The family to which each trematode species belonged was obtained from the taxonomic classification in Gibson et al. (2002), Jones et al. (2005), and Bray et al. (2008). Mean monthly air temperatures for each locality were obtained from NOAA’s GHCN_CAMS Gridded Land Temperature database (https://psl.noaa.gov) based on the coordinates and year of sampling (Fan and Dool 2008). In cases where infection data were only given pooled by season, we averaged the monthly mean temperatures by season (for example, averaging July and August for northern summer). Since water temperatures were not available, we assumed that local air temperatures were a reasonable proxy for water temperature.

### Data analyses

Based on the biology of trematodes, we split our dataset into two for analysis. The first include all entries in which the host sampled acted as first intermediate host (always a mollusc) for the trematode parasite; in these cases, the only measure of infection that makes sense is prevalence. The second data subset included the remaining entries, for which we used both prevalence and abundance as measures of infection. If abundance was not given in the original studies, it was computed as: abundance = prevalence × intensity, where possible.

Our goal was to determine to what extent the local thermal regime drives the seasonal change in trematode infections from spring onward, and whether this effect depends on the parasite’s life cycle stage or other aspects of the local system. For this purpose, we computed a single estimate of the seasonal change in infection level ($\Delta P$ or $\Delta A$) for each entry in our dataset, as the difference between infection levels in the spring and at the peak of summer:

$$\Delta P=J\left({P}_{su}-{P}_{sp}\right)$$

$$\Delta A=J\left({A}_{su}-{A}_{sp}\right)$$

$$J=1-\frac{3}{4\left({N}_{su}+ {N}_{sp}-2\right)-1},$$

where ${P}_{su}$ and ${P}_{sp}$ are the prevalence of infection in summer and spring, respectively; ${A}_{su}$ and ${A}_{sp}$ are the abundance of infection in summer and spring, respectively; ${N}_{su}$ and ${N}_{sp}$ are the host sample sizes in summer and spring, respectively; and $J$ is a correction for small sample sizes. This correction is the same as that long recommended for calculations of effect sizes (Hedges and Olkin 1985); it leads to a slight reduction of the $\Delta$ values when sample sizes are small, producing more conservative estimates. Positive $\Delta P$ or $\Delta A$ values indicate that infections increased from spring to summer, whereas negative values indicate the opposite; the greater the value, the more pronounced the change.

For entries in which infection data were given on a month-by-month basis, we considered April and May as spring, and July and August as summer in studies from the northern hemisphere (October and November as spring and January and February as summer in the southern hemisphere). When data for each month were available, values for each pair of months were averaged, as were their sample sizes; when data were only available for one of those months, we used that instead. Both the month-by-month and seasonal datasets were merged into a single dataset prior to analysis. Some entries in the dataset had to be excluded from analysis because of missing data for the focal months, or (in one case) extreme outlier values.

We analysed the data using three generalised linear mixed models (GLMM) with Gaussian distribution, one for the data subset on infections of first intermediate hosts with $\Delta P$ as response variable, and two for the data subset on infections of other hosts (second intermediate or definitive) with $\Delta P$ and $\Delta A$ as response variables. The GLMMs were implemented using the *lme4* package (Bates et al. 2015) in the R computing environment (version 4.1.2; R Core Team 2021). In each GLMM, the main predictor was the difference in mean temperature between spring and the peak of summer, $\Delta T$, calculated as the $\Delta$ values above except for the $J$ correction. The habitat type (pond, river, or lake) was also included as a categorical predictor. Two further predictors were included in the analyses of infections of second intermediate or definitive hosts with $\Delta P$ and $\Delta A$ as response variables: the role of the host in the life cycle (second intermediate host or definitive host), and the host higher taxon (mollusc, crustacean, leech, amphibian, or fish). In all GLMMs, we also incorporated two random factors: the family to which the trematode species belonged, to account for possible phylogenetic effects, and the article from which the data originated, since some articles contributed two or more entries and thus to account for any potential non-independence among entries.

## Results

Overall, our dataset comprised 782 estimates of seasonal (spring-to-summer) change in trematode infection in freshwater hosts (see full dataset in Supplementary Table S2). The trematode species included belonged to 39 families, the ones with the most species being Echinostomatidae and Diplostomidae. All first intermediate hosts were molluscs, whereas fish and molluscs were the most common second intermediate hosts, and all definitive hosts were fish. In about half of all entries, the habitat type was a lake, with rivers being the second most common habitat types. The vast majority of entries were from the northern hemisphere.

**Fig. 1:**
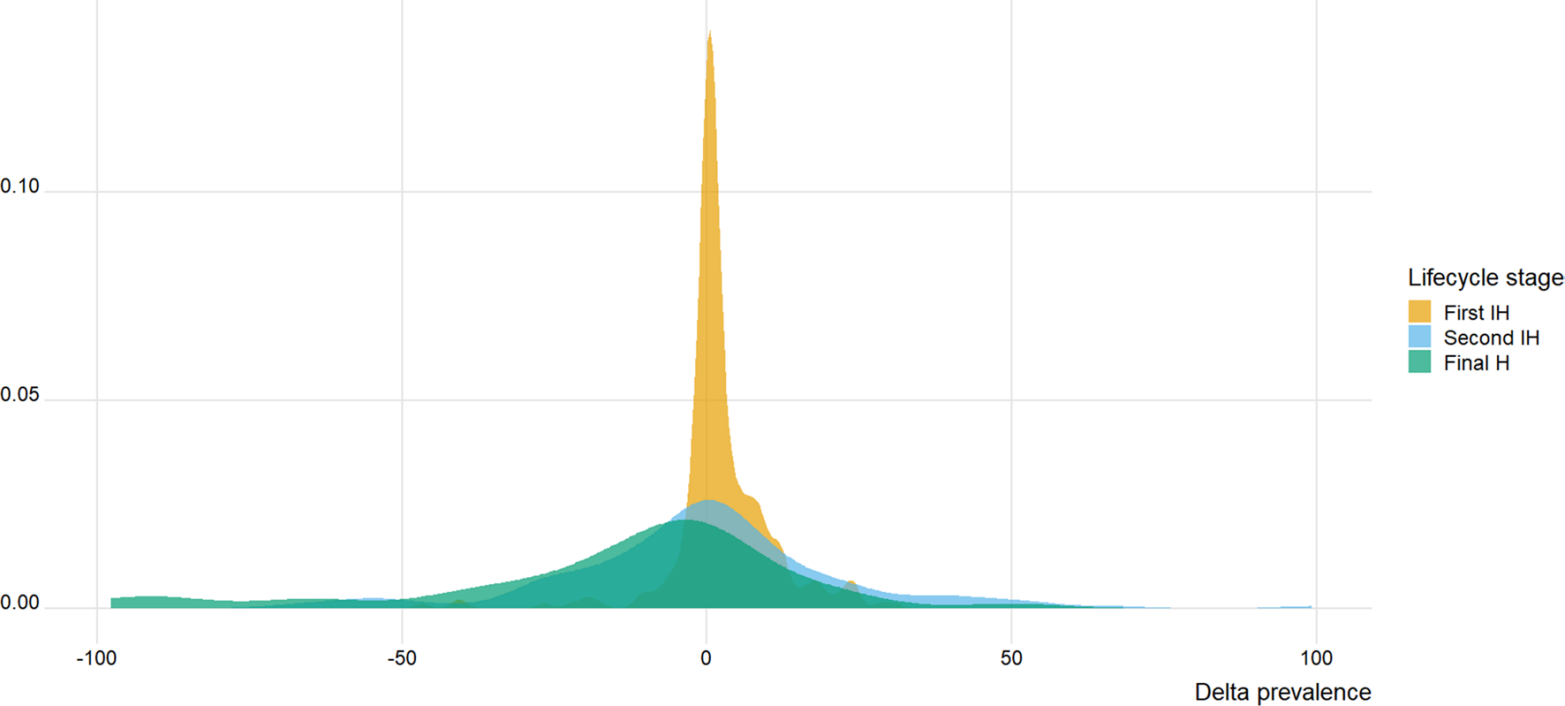
Frequency distribution of delta prevalence ($\Delta P$) values, corresponding to the difference in prevalence from spring to summer, for trematode infections of first intermediate hosts (First IH; *N* = 278), second intermediate hosts (Second IH; *N* = 147), and definitive hosts (Final H; *N* = 84) in temperate freshwater ecosystems

**Fig. 2:**
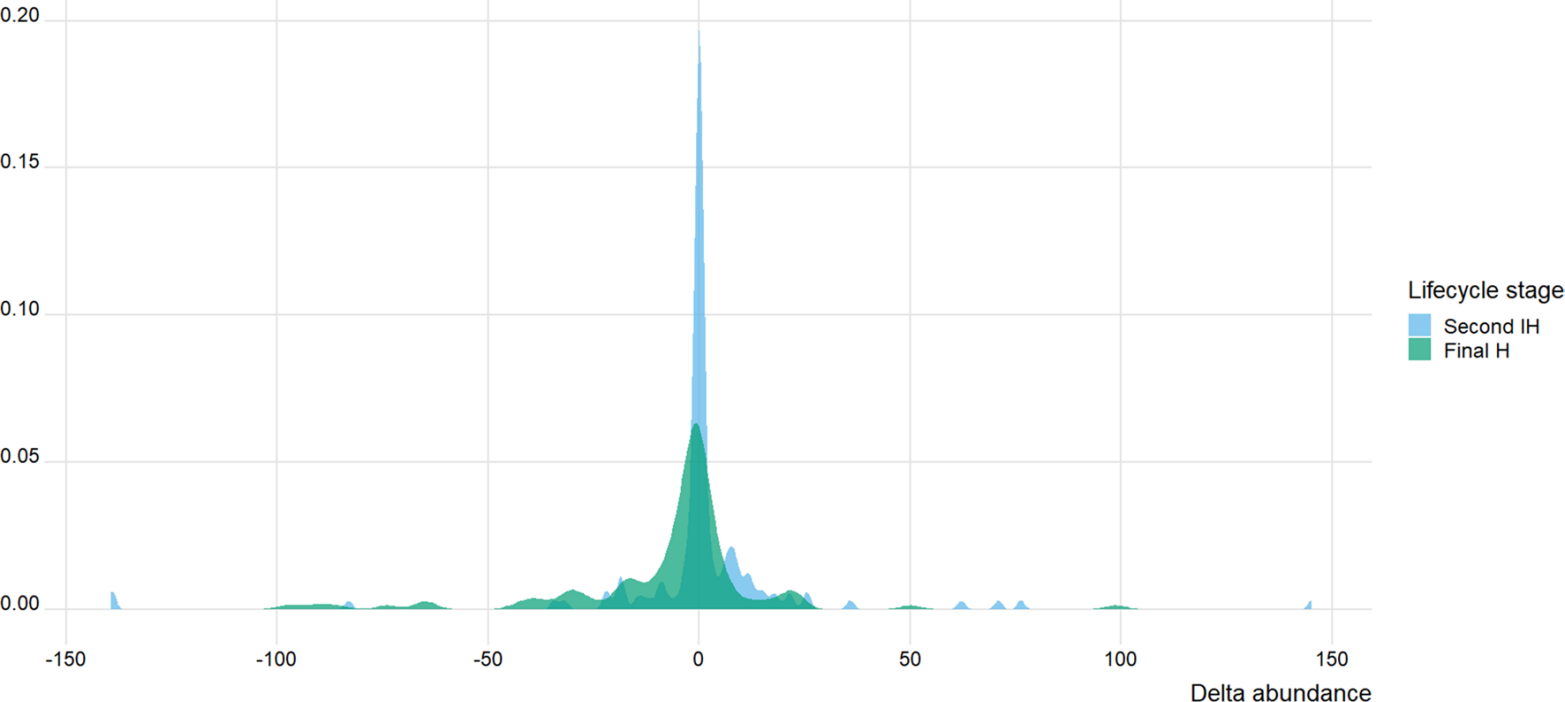
Frequency distribution of delta abundance ($\Delta A$) values, corresponding to the difference in abundance from spring to summer, for trematode infections of second intermediate hosts (Second IH; *N* = 139) and definitive hosts (Final H; *N* = 112) in temperate freshwater ecosystems

All estimates of spring-to-summer change in temperature, $\Delta T$, were positive, ranging from 1.8 °C to 18 °C. In contrast, infection levels were not consistently reported as rising from spring to summer, with a high frequency of negative $\Delta P$ or $\Delta A$ values (Figs. 1 and 2). Indeed, negative $\Delta P$ values (seasonal decrease in prevalence) were observed for 74 (27%) out of 278 entries for first intermediate hosts, 70 (48%) out of 147 entries for second intermediate hosts, and 56 (67%) out of 84 entries for definitive hosts. Similarly, negative $\Delta A$ values (seasonal decrease in abundance) were observed for 66 (47%) out of 139 entries for second intermediate hosts, and 74 (66%) out of 112 entries for definitive hosts.

In absolute terms, i.e., ignoring whether they are positive or negative, spring-to-summer changes in trematode prevalence, $\Delta P$, were generally rather small for first intermediate hosts, whereas much larger values were often observed for second intermediate hosts and definitive hosts (Fig. 1). Similarly, absolute values of spring-to-summer changes in trematode abundance, $\Delta A$, were a little more likely to be large for definitive hosts than for second intermediate hosts (Fig. 2).

**Fig. 3:**
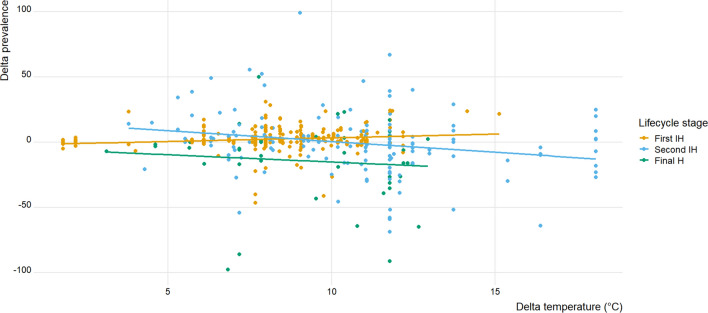
Delta prevalence ($\Delta P$) values, corresponding to the difference in prevalence from spring to summer, plotted against delta temperature ($\Delta T$) values, for trematode infections of first intermediate hosts (First IH; *N* = 278), second intermediate hosts (Second IH; *N* = 147), and definitive hosts (Final H; *N* = 84) in temperate freshwater ecosystems. Trend lines are shown for illustrative purposes; only the relationship for first intermediate hosts is significant

Results of GLMMs are summarised in Table 1. Our first GLMM revealed that the spring-to-summer change in temperature, $\Delta T$, had a weak but significant (analysis of deviance: *P* = 0.034) positive effect on the spring-to-summer change in prevalence of infection, $\Delta P$, in first intermediate hosts (Fig. 3; Table 1). The type of habitat (lake, pond or river), however, had no influence on seasonal changes in trematode prevalence in first intermediate hosts.

**Table 1:** Results of the three generalised linear mixed models testing the effects of different predictors (seasonal temperature change, habitat type, host role in the life cycle, and host taxon) on seasonal change in trematode infection levels

Predictor	Estimate	Standard error	95% confidence intervals	*t*-value
Prevalence ($\Delta P$) in first intermediate hosts (*N* = 278)				
(Intercept)	− 1.203	2.924	− 6.705, 4.312	0.411
Seasonal temperature change ($\Delta T$)	**0.635**	**0.282**	**0.098, 1.168**	**2.255**
Habitat type: pond	− 2.281	1.801	− 5.736, 1.113	1.267
Habitat type: river	− 0.832	2.133	− 4.883, 3.203	0.390
Prevalence ($\Delta P$) in second intermediate and definitive hosts (*N* = 231)				
(Intercept)	3.347	22.597	− 40.492, 44.876	0.148
Seasonal temperature change ($\Delta T$)	− 0.945	0.771	− 2.397, 0.475	1.226
Habitat type: pond	12.625	8.165	− 2.729, 28.064	1.546
Habitat type: river	5.860	4.667	− 2.855, 14.673	1.255
Host life cycle role: second intermediate	2.110	5.610	− 11.606, 15.032	0.376
Host taxon: crustacean	− 4.987	22.947	− 47.436, 37.454	0.217
Host taxon: fish	− 2.309	20.350	− 40.056, 37.457	0.113
Host taxon: leech	8.047	25.165	− 37.811, 54.899	0.319
Host taxon: mollusc	0.513	20.601	− 37.546, 38.868	0.025
Abundance ($\Delta A$) in second intermediate and definitive hosts (*N* = 251)				
(Intercept)	6.806	25.402	− 42.371, 56.477	0.268
Seasonal temperature change ($\Delta T$)	0.424	0.816	− 1.217, 1.928	0.519
Habitat type: pond	− 2.370	9.610	− 20.491, 16.130	0.247
Habitat type: river	5.310	4.521	− 3.250, 14.241	1.174
Host life cycle role: second intermediate	− 12.973	8.845	− 31.933, 9.079	1.467
Host taxon: crustacean	2.787	25.191	− 44.671, 48.851	0.111
Host taxon: fish	− 18.749	22.238	− 60.781, 24.608	0.843
Host taxon: leech	− 12.553	26.250	− 63.013, 38.892	0.478
Host taxon: mollusc	1.409	24.510	− 44.456, 46.250	0.057

For categorical predictors, the reference level included in the intercept is ‘lake’ for Habitat type, and ‘amphibian’ for Host taxon; alternate analyses with other reference levels yielded similar results

Significant effects (95% confidence intervals not overlapping zero) are shown in bold

**Fig. 4:**
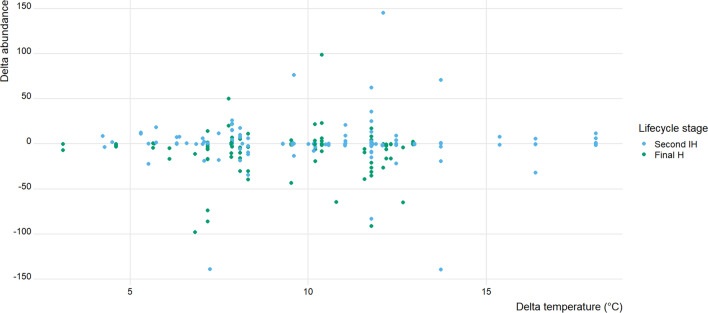
Delta abundance ($\Delta A$) values, corresponding to the difference in abundance from spring to summer, plotted against delta temperature ($\Delta T$) values, for trematode infections of second intermediate hosts (Second IH; *N* = 139) and definitive hosts (Final H; *N* = 112) in temperate freshwater ecosystems

In contrast, in other hosts in the trematode life cycle, spring-to-summer changes in either prevalence, $\Delta P$, or abundance of infection, $\Delta A$, were not related to local spring-to-summer change in temperature, $\Delta T$ (Figs. 3 and 4; Table 1). None of the other factors investigated, i.e., habitat type, host taxon, or whether the host acted as second intermediate host or definitive host, influenced seasonal changes in trematode infection (Table 1).

## Discussion

Disease dynamics often show strong seasonality patterns (Altizer et al. 2006). In addition to temperature influencing the physiology and reproduction of parasites within ectothermic hosts, abiotic conditions also play a major role in determining the survival and transmission success of parasites’ free-living stages outside the host (Pietrock and Marcogliese 2003), providing a direct mechanistic link between seasonal conditions and infection risk. Following earlier qualitative syntheses of seasonality patterns of trematode infections in freshwater systems (e.g., Chubb 1979), we present the first large-scale quantitative test of general seasonal patterns of infection by trematodes at all stages of their life cycle, and an assessment of plausible drivers of variation in their seasonal responses. What our analysis uncovers is a surprisingly high degree of variability among host–parasite systems, and a minimal effect of seasonal rise in temperature from spring to summer on concurrent changes in infection levels by trematodes.

Indeed, prevalence of infection in snail first intermediate hosts was observed to decline from spring to summer in about one-quarter of the freshwater host–parasite systems investigated. Prevalence and abundance of infection by trematodes in their second intermediate hosts decreased in about half of the systems investigated; for definitive hosts, this decline occurred in two-thirds of systems investigated. There is, therefore, no universal pattern of rising infections as temperature increases seasonally. Spring-to-summer declines in infections levels are commonly observed, suggesting that system-specific idiosyncrasies prevent any generalisation.

Only one significant pattern emerged from our analysis: the greater the rise in temperature from spring to summer, the greater the rise in prevalence of trematode infections in snail intermediate hosts. Although weak, this trend may be explained by general responses of trematodes to thermal conditions. For instance, both the per capita egg output by adult trematodes in ectothermic definitive hosts and hatching success of those eggs are temperature-dependent, reaching their peak at higher temperature though declining at extreme temperatures (Morley and Lewis 2017). Thus, the number of infective stages released into the aquatic environment may rise in proportion to ambient temperatures, at least up to a point. Similarly, the infectivity of miracidia hatched from trematode eggs to their snail first intermediate hosts also increases with rising temperatures, again up to a point beyond which it plateaus then drops (Morley and Lewis 2015). However, our finding may be due, to some extent, to the fact that infected snails are easier to identify in summer. Indeed, most temperate freshwater snails have an annual life history: after overwintering, adults reproduce in early spring, producing a cohort of new recruits. The new infections acquired by these young snails early in their life will take time to develop within their tissues. Since most studies rely on the visual detection of trematode stages (sporocysts, rediae) within molluscs and because the asexual proliferation of these stages has long been known to ramp up at higher temperatures (e.g., Stirewalt 1954; Schell 1962), confirming that a snail is infected may be easier in summer, whereas early pre-patent infections in spring might be overlooked. Nevertheless, our findings do suggest that a slightly greater proportion of many freshwater snail populations is infected by trematodes in mid-summer than during spring, depending on the magnitude of the seasonal temperature increase.

The temperature-driven spring-to-summer rise in infections of snail first intermediate hosts was not mirrored by a rise in infections of second intermediate hosts. This was true whether the second intermediate host was an invertebrate or vertebrate, and whether they lived in rivers, ponds (small, well-mixed, often temporary lentic habitats) or lakes (larger, permanent, often stratified lentic habitats). This is a somewhat paradoxical result, as a greater proportion of snail first intermediate hosts releasing cercariae in summer should raise the risk of infection for the next host in the life cycle. Furthermore, higher temperatures have been repeatedly shown to increase the output of cercariae per infected snail (Poulin 2006) and the infectivity of cercariae to the next host (Morley and Lewis 2015). However, studies of the thermal sensitivity of cercariae with respect to their production within the snail first intermediate host and their subsequent survival and infectivity have generally been conducted under controlled laboratory conditions (e.g., Paull et al. 2012; Selbach and Poulin 2020; Taskinen et al. 2022). In contrast, the data analysed in the present study were collected under natural conditions. The differences between the artificial conditions of the laboratory, where the effect of temperature can be singled out, and those of natural habitats where a myriad other factors can also act on trematodes, may explain the discrepancies between our findings and those of experimental studies. The effect of temperature may be clear-cut in the latter, but obscured by confounding factors in nature. One of these confounding factors may be predation on cercariae by non-host organisms (Johnson et al. 2010; Mironova et al. 2020; Koprivnikar et al. 2023), which may intensify at high summer temperatures (e.g., Goedknegt et al. 2015) and offset the higher output of cercariae from first intermediate hosts (but see Gopko et al. 2020). Another possibility is that parasite-induced mortality may be exacerbated by higher temperatures, leading to the disappearance of the more heavily infected second intermediate hosts (see Gordon and Rau 1982; Rousset et al. 1996; Fredensborg et al. 2004) and thus lowering observed mean abundance of infection in summer. Heavy infections do not have to cause direct host mortality, they could also indirectly lead to the removal of the more heavily infected hosts in summer via behavioral alterations that increase host susceptibility to predation (Poulin 2010). These and other possible factors (e.g., stronger host immune responses at higher summer temperatures) could counteract the greater output of cercariae in summer in many ecosystems, and lead to system-specific net effects of temperature on trematode infections in second intermediate hosts. Outdoor mesocosm experiments, re-creating more complex natural communities and conditions than those in a simple two-player, host-parasite laboratory study, would be a promising way forward to assess the impact of seasonal temperature rise on trematode infection patterns.

Similarly, several possible scenarios can explain the lack of spring-to-summer rise in infection levels in definitive hosts. For example, even if prey consumption rates by fish increase with temperature (e.g., Hill and Magnuson 1990), a summer shift to different microhabitats or to other preferred prey may limit their ingestion of infected second intermediate hosts during summer. Furthermore, as most fish species live more than 1 year, their trematode burden at any one time reflects accumulation across more than a single spring-to-summer period. The lack of clear seasonal trend in infection levels of fish definitive hosts seen here parallels some of the findings of an earlier study with a different dataset and slightly different focus (Poulin 2020).

Overall, our findings suggest that the thermal optimum for within-host processes, such as asexual multiplication in the first intermediate host, and that for epidemiological processes at the host population level (e.g., infection rates) may not be the same. Indeed, there is evidence that temperature dependence is decoupled between individual and population levels in parasites transmitted from the environment by infective stages (Kirk et al. 2022). Our study has some limitations, however. Our data come from studies that compare snapshots of infection levels across different months or seasons, and therefore, they do not necessarily capture temporal dynamics in parasite acquisition. Too few studies actually quantified seasonal changes in the probability of gaining parasites per unit time (e.g., Wetzel and Esch 1997) for us to use their data. Other studies deployed sentinel snails, i.e., uninfected snails held in situ within cages from which they can be recaptured, to estimate temporal changes in rates of infection (e.g., Ménard and Scott 1987). Again, too few studies adopted this approach for any meaningful analysis. Such field studies using sentinel hosts, replicated in time and space, would be a powerful approach to quantify seasonal changes in trematode transmission dynamics and infection risk. Nevertheless, the data we used can provide an indirect but representative picture of rises and falls in infection risk across seasons.

Moreover, we estimated the temperature experienced by aquatic hosts based on overall (monthly) mean values of air temperature at the given coordinates during sampling. The correspondence between local air and water temperatures is generally good, but not always perfect (Armitage 2023). Furthermore, the temperature and seasonal changes experienced by the organisms in their aquatic environment might have differed among individuals even when sampled at the same localities, e.g., organisms in shallow littoral regions vs. organisms at greater depth of a lake. Nevertheless, on the very large scale at which our analysis was conducted, small deviations between the temperature estimates we used and the temperatures experienced by focal organisms are probably negligible.

Natural selection has optimized the timing of key life history events in response to predictable seasonal changes in temperature, daylight, and other abiotic factors (Forrest and Miller-Rushing 2010; Varpe 2017; Park and Post 2022). The synchronisation of host phenology with seasonal environmental changes has consequences for parasites, resulting in adaptations that improve their survival and transmission (Starkloff and Civitello 2022). Several recent reviews have summarised various ways in which climate change can impact parasitism and disease (e.g., Marcogliese 2001, 2016; Byers 2020; Rohr and Cohen 2020). Our findings suggest that if host phenology shows adaptive responses to changing climatic conditions, the indirect consequences for trematode infection levels will not be easy to predict across different systems, as they appear decoupled from the general phenological and successional patterns observed in freshwater ecosystems.

Against a backdrop of mostly predictable phenological and successional patterns characterising free-living aquatic organisms across different temperate freshwater systems, from phytoplankton to fish (Sommer et al. 2012), trematode infections display mostly unpredictable, idiosyncratic seasonal changes among different species. Earlier studies on particular systems had revealed much interspecific variation in trematode responses to rising temperatures, even among related and/or sympatric trematode species using the same host species (Selbach and Poulin 2020; Born-Torrijos et al. 2022; Taskinen et al. 2022). Our analysis confirms this on a global scale. This variability is not unique to trematodes (e.g., Finn et al. 2022). Our results indicate that there is no universal pattern of rising trematode infections in summer, for any host in their life cycle; instead, seasonal variation in infection appears species- or system-specific, preventing any broad generalisation. This compounds the challenges already pointed out by Woods et al. (2022) for future research into phenology and community succession in freshwaters, if we are to anticipate and mitigate future risks to ecosystem functioning under a changing climate.

### Supplementary Information

Below is the link to the electronic supplementary material.

Supplementary file1 (XLSX 1544 KB)

## Data Availability

All data used in the analyses in this study are available in Supplementary Table S2.
